# Combined Immunofluorescence (IFA) and Fluorescence In Situ Hybridization (FISH) Assays for Diagnosing Babesiosis in Patients from the USA, Europe and Australia

**DOI:** 10.3390/diagnostics10100761

**Published:** 2020-09-28

**Authors:** Jyotsna S. Shah, Eddie Caoili, Marie Fe Patton, Snehal Tamhankar, Mu Mu Myint, Akhila Poruri, Olivia Mark, Richard I. Horowitz, Alan D. Ashbaugh, Ranjan Ramasamy

**Affiliations:** 1 ID-FISH Technology Inc., Milpitas, CA 95035, USA; 2 IGenex Inc., 556 Gibraltar Drive, Milpitas, CA 95035, USA; ecaoili@igenex.com (E.C.); mpatton@igenex.com (M.F.P.); stamhankar@igenex.com (S.T.); mmyint@igenex.com (M.M.M.); aporuri@igenex.com (A.P.); omark@igenex.com (O.M.); 3 Hudson Valley Healing Arts Center, New York, NY 12538, USA; medical@hvhac.com; 4 HHS Subcommittee on Babesia and Tick-Borne Pathogens, US Department of Health and Human Services, Washington, DC 20201, USA; 5 College of Medicine, University of Cincinnati, Cincinnati, OH 45221, USA; ashbauad@ucmail.uc.edu

**Keywords:** *Babesia duncani*, *Babesia microti*, babesiosis, immunofluorescence assay, fluorescence in situ hybridization, laboratory diagnosis of babesiosis

## Abstract

Apicomplexan parasites of the genus *Babesia* cause babesiosis in humans and animals worldwide. Human babesiosis is a predominantly zoonotic disease transmitted by hard ticks that is of increasing health concern in the USA and many other countries. Microscopic examination of stained blood smears, detection of serum antibodies by immunoassays and identification of parasite nucleic acid in blood by qPCR and fluorescence in situ hybridization (FISH) are some methods available for diagnosing babesiosis. This study investigated the use of a *Babesia* genus-specific FISH test for detecting *Babesia* parasites in blood smears and immunofluorescence assay (IFA) for detecting serum antibodies to *Babesia duncani* and *Babesia microti*, two common species that cause human babesiosis in the USA. The findings with clinical samples originating from USA, Australia, Europe and elsewhere demonstrate that the parallel use of *Babesia* genus-specific FISH and IFA tests for *B. duncani* and *B. microti* provides more useful diagnostic information in babesiosis and that *B. duncani* infections are more widespread globally than presently recognized.

## 1. Introduction

Apicomplexan protozoan parasites of the genus *Babesia* cause babesiosis in humans and animals [1,2,3]. There were 2161 cases of human babesiosis reported in the USA in 2018 to the US Centers for Disease Control and Prevention (CDC) [4]. *Ixodes scapularis*, *I. ricinus*, *I. persulcatus* and *Dermacentor albipictus* are some hard ticks that transmit babesiosis to humans after acquiring *Babesia* species from reservoir animals such as white-footed mice and mule deer [3,4,5]. Human to human transmission of *Babesia* species can occur through blood transfusion, congenital transmission and organ transplantation [3,4,6]. *Babesia microti*, *B. duncani* and *B. divergens* are mainly responsible for human babesiosis in the USA [2,3,4] with *B. microti* and *B. duncani* considered to be, respectively, more prevalent in the East and West coasts of North America [5,7,8]. *Babesia microti*, *B. divergens*, *B. venatorum* and *B. crassa* are responsible for babesiosis in Eurasia [2,3]. Babesiosis is also prevalent in Africa, Australia and South America [2,3].

The two main approaches for diagnosing human babesiosis in a clinical laboratory are the detection of parasites in blood and assaying antibodies produced against the parasite.

Parasites in peripheral blood are frequently detected by examining stained blood smears by microscopy. However this method cannot identify *Babesia* parasites at the species level. Alternatively, *Babesia* parasite nucleic acid is detected by qPCR on blood samples [3,4] and the detection of ribosomal RNA within infected red blood cells (iRBCs) by fluorescence in situ hybridization (FISH) [9]. Several qPCR tests have been developed for *B. microti* and are reported to detect <10 parasites per μL of blood [10,11]. They are the preferred methods for screening blood for transfusion [12,13]. Although a qPCR test for *B. duncani* has been recently developed [14], it is not yet in common use. The detection of *B. duncani* and *B. microti* in blood with the *Babesia* genus-specific FISH has been estimated to have a limit of detection of 58 parasites per μL of blood and provides highly specific laboratory confirmation of babesiosis [9]. Despite a lower sensitivity of detection, the *Babesia* genus-specific FISH test has several advantages, including lower resource and shorter time requirements, over qPCR tests, and these have been previously discussed in detail [9]. However, *Babesia* parasite concentrations in peripheral blood can be low very early in an infection and during chronic low grade infections where parasites may be sequestered by binding to capillary endothelia in internal organs. Cytoadherence to the capillary endothelium has been reported in *B. duncani* [15], but cytoadherence and the variant antigens on the surface of infected red blood cells (iRBCs) that are responsible for it have been better characterized in the bovine parasite *Babesia bovis* [16]. Sequestration in the microvasculature is also a strategy adopted by the closely-related apicomplexan malaria parasite *Plasmodium falciparum* to avoid destruction while circulating through the spleen and is mediated by a family of *var* genes that code for variant antigens termed PfEMPs expressed on the surfaces of iRBCs [17].

Serum antibodies are commonly detected by immunofluorescence assays (IFA) performed with *B. microti* fixed on microscope slides [18,19], but an equivalent IFA has not been widely used for detecting antibodies against *B. duncani*. An ELISA utilizing recombinant proteins as antigens that has been recently developed for *B. microti* is less sensitive than IFA [20] and is not yet in common use for diagnosis. There is presently no report of an ELISA test for *B. duncani*. Immunochromatography-based lateral flow tests have been recently trialed for point-of-care diagnosis of bovine babesiosis [21], but similar tests are not yet available for human babesiosis. IgM is the first antibody class to be formed in a primary immune response. IgM antibodies are produced early, usually within days, during an infection before class switching later to higher affinity IgG and other immunoglobulin classes. Serum antibodies may therefore be below the threshold of detection in the very early stages of an infection. As the infection resolves, either as a result of the immune response or through drug treatment, antibody levels begin to diminish but can persist at detectable levels for several months. A total immunoglobulin or IgG IFA titer of ≥1:256 is recognized by the CDC as laboratory evidence that supports a diagnosis of babesiosis [22]. IgM IFA titers of ≥1:32 have been, however, reported to have high sensitivity and specificity for acute or early *Babesia* infections [23]. Detection of anti-*Babesia* antibodies per se does not differentiate between an active or ongoing infection and a resolved past infection, although high IgG antibody titers indicate a probable active indication [22]. A marked increase in IFA titers over time in a patient is a better indicator of an active infection, but the required temporal follow-up in serum collection and testing is often not easily possible.

Chronic babesiosis can be symptomatic or asymptomatic and occur in immunocompromised patients, the elderly and some immunocompetent patients [24]. The biological basis for such persistence has not been well studied but may involve multiple immune evasion mechanisms employed by *Babesia* parasites analogous to the mechanisms that have been better studied in malaria parasites [17], including the recently identified roles for iRBC surface-expressed variant proteins of the rifin family in impairing protective immune responses in malaria [25]. Additionally, mutations in the *cytb* and *rpl4* genes of *B. microti* governing atovaquone and azithromycin resistance, which is now commonly seen in clinical practice, can also contribute to chronic babesiosis [26,27]. Chronic babesiosis accompanied by immune evasion poses diagnostic problems because of low peripheral blood parasitemias, antibodies that may not markedly switch from IgM to IgG [28] and antibody levels that may remain low and show little temporal variation.

It is in this context that we report findings from the concomitant use of the recently developed *Babesia* genus-specific FISH test for detecting *Babesia* parasites in the blood [9] and two indirect IFA tests for separately detecting IgM and IgG antibodies against both *B. duncani* and *B. microti* for the laboratory diagnosis of babesiosis. The tests were performed on clinical blood samples received from the USA, Australia and Europe for routine testing for babesiosis and had the added advantage of being able to determine the relative prevalence of *B. duncani* and *B. microti*.

## 2. Materials and Methods

### 2.1. Clinical Blood Samples for Parallel IFA and FISH Tests

IFA tests for *B. duncani* and *B. microti* and the *Babesia* genus-specific FISH test were performed on serum and EDTA-treated blood, respectively, from 390 patients that were received by IGeneX for routine testing for tick borne diseases and babesiosis. These were composed of 249 samples from different states in the USA (including one from the USA territory of Puerto Rico in the Caribbean) received in 2018 as well as 89 from Europe, 49 from Australia and one each from Antigua and Barbuda, India and Singapore received in 2016 and 2017.

### 2.2. Indirect Immunofluorescence Assays (IFA)

Smears on microscope slides prepared from the blood of hamsters infected with *B. duncani* (ATCC PRA-302) and *B. microti* (ATCC 30221D), provided by Dr. Alan Ashbaugh, University of Cincinnati, OH, USA, were used in IFA assays for detecting antibodies in clinical sera essentially as previously described [18,19] but with modifications for separately detecting IgM and IgG antibodies. All samples were first screened at 1:20 dilution of serum in 0.01 M phosphate buffered saline at pH 7.2 (PBS). Any sample that was positive in this screening test was serially diluted by two-fold dilutions up to 1:1028 in PBS for further testing. For tests, a 25 μL aliquot of diluted serum was added to a slide well. Each slide was incubated for 30 min at 37 ℃, followed by three washes with PBS at ambient temperature. Then 25 μL of goat anti-human IgG or IgM immunoglobulin labeled with DyLightTM 488 (SeraCare, Milford, MA, USA diluted at 1:800 in PBS and 0.0005% Evans Blue was added to each well, and the slides were incubated for 30 min at 37 ℃. The slides were then washed three times with PBS, air dried, mounted with 4–5 drops of mounting medium (Scimedex, Denville, NJ, USA) and overlaid with a coverslip. Fluorescence in slides was viewed in a fluorescence microscope (Olympus, Tokyo, Japan) at 400× magnification. Antibody controls used in every test were serum from a patient with babesiosis (positive control), serum from a healthy subject (negative control) and PBS. Based on optimal sensitivity and specificity of detection, indirect IFA staining of parasites at serum dilutions of 1:20 and 1:40 were considered borderline positive and >1:20 and >1:40 as positive for IgM and IgG anti-*Babesia* antibodies respectively at IGeneX.

### 2.3. Babesia Genus-Specific FISH Assay

The *Babesia* genus-specific FISH assay was performed on blood smears on glass microscope slides using a kit (catalogue number BabGK04 from ID-FISH Technology Inc., Milpitas, CA, USA) according to the manufacturer’s instructions as described previously in detail [9]. Fluorescence was then viewed using a light microscope with an LED attachment containing 492 nm excitation and 530 nm emission band pass filters (ID-FISH, Milpitas, CA, USA), as described [9].

### 2.4. Specificity Controls for the B. duncani and B. microti IFA Tests with Sera from Associated Diseases

Sera received for testing and found to be positive at IGeneX for three other tick-borne diseases and *Bartonella hensalae* infection were used as specificity controls for the *B. duncani* and *B. microti* IFA tests. These were composed of 20 sera positive for antibodies with titers ≥1:40 in a Lyme IFA test and positive by a Lyme immunoblot assay for infection with *Borrelia burgdorferi s.s.* [29]; eight sera positive in an IFA test for *Ehrlichia chaffeensis* causing human monocytic ehrlichiosis; 12 sera positive in an IFA test for *Anaplasma phagocytophilum* causing human granulocytic anaplasmosis, all with IgG titers of 1:80–1:640, including two with IgM titers of 1:160 and 1:640; and 20 sera positive in an IFA test for *Bartonella hensalae* infections composed of 15 sera with IgG titers of 1:160 or 1:320 and five sera with IgM titers of 1:20–1:80. Details of the IGeneX IFA tests for anaplasmosis, ehrlichiosis and bartonellosis are available at www.igenex.com (accessed on 16 September 2020).

### 2.5. Ethics Statement

Retrospective analysis of de-identified clinical test results and use of leftover de-identified sera that would otherwise have been discarded do not require institutional review board approval or consent from patients in the USA. Infection of hamsters with *Babesia* parasites was approved by the IACUC at the University of Cincinnati (A3446-01 of 20 November 2018).

## 3. Results

### 3.1. Specificity of the B. duncani and B. microti IFA Assays Tested with Sera Positive for Antibodies in Bartonellosis, Ehrlichiosis and Lyme Borreliosis

The twenty sera from patients with bartonellosis, 20 from patients with either human monocytic ehrlichiosis or human granulocytic anaplasmosis and 20 from patients with Lyme borreliosis showed no reaction for either IgG or IgM antibodies in the *B. duncani* and *B. microti* IFA tests at a serum dilution of 1:20.

### 3.2. Parallel IFA and FISH Tests Performed on Clinical Blood Samples

Findings from the 249 USA samples in the *B. duncani* and *B. microti* IFA tests and the *Babesia* genus-specific FISH test are summarized in Table 1.

Details of the IFA and FISH test results from samples originating in the different USA states and the Caribbean territory of Puerto Rico are shown in Supplementary Figure S1. California had 23 and 5 samples that were, respectively, positive in the *B. duncani* and *B. microti* IFA tests. More samples were positive for *B. duncani* than *B. microti* IFA test in the five Mid-West states. The 12 East coast states had a more even distribution of samples that were positive in the *B. duncani* and *B. microti* IFA tests. The one Puerto Rican sample was only positive in the *B. duncani* IFA test.

Of the 89 USA samples that were shown positive for babesiosis by either FISH or IFA, 53 (60%) were positive in the IFA tests for *B. duncani* and *B. microti* but negative in the *Babesia* genus-specific FISH test. Twenty one of these 53 patients (40%) had IgG IFA titers of ≤1:80 and were interpreted as suggestive of antibodies remaining after the resolution of an infection.

Of the 36 samples that were identified as positive in the *Babesia* genus-specific FISH test, 31 (86%) showed positive reactions in either or both of the *B. duncani* and *B. microti* IFA tests. Details of the IFA reactivity of the 36 FISH positive samples are presented in Table 2. All 31 samples had relatively high IFA titers for IgM antibodies of 1:40–1:160 and were either negative or had titers of ≤1:320 for IgG. Two samples marked with asterisks that had titers of 1:160 for IgM in the *B. microti* IFA test also had titers of 1:160 and 1:320 for IgG in the *B. duncani* IFA test, interpreted as characterizing probable active infection with *B. microti* and either a probable active infection or resolving infection with *B. duncani*.

Detailed results of the FISH and IFA tests performed on samples from Australia and Europe as well as two samples from Asia and one other sample from a Caribbean island are provided in Supplementary Figure S2. The results provide evidence for babesiosis caused by *B. duncani* in Queensland, New South Wales, Victoria and South Australia as well as Ukraine, Antigua and Barbuda, and India. The results identified *B. microti* infections in the UK and Ukraine in Europe as well as New South Wales and Victoria in Australia. A summary of the findings from Australian and European samples are presented in comparison to those from the USA in Table 3. They suggest that approximately 31.5% of patients tested worldwide for suspected babesiosis or tick-borne diseases have been infected with *Babesia* parasites.

## 4. Discussion

Microscopic examination of stained blood smears for parasites and IFA tests on fixed parasites are two readily available and commonly recognized laboratory methods for diagnosing babesiosis [3,4,18,19]. The qPCR test requiring amplification of DNA from parasites in blood is highly sensitive but requires stringent controls and sophisticated laboratory resources and is therefore suitable for screening large numbers of blood samples for the purpose of blood transfusion [12,13]. The *Babesia* genus-specific FISH test has many advantages that make it more appropriate for diagnostic use with the relatively smaller numbers of samples that require testing for tick-borne disease in small clinical laboratories [9]. Furthermore, an independent study demonstrated 96% agreement between a qPCR and the *Babesia* genus-specific FISH test for diagnosing babesiosis [30]. IFA and FISH detect antibodies and parasites, respectively, and therefore constitute complementary methods for diagnosing babesiosis in small laboratories. The FISH test detects active infection but may not detect low peripheral blood parasitemias at very early stages of an infection, chronic late-stage infections involving parasites sequestration in tissues or resolving infections. On the other hand IFA detects antibodies that are usually produced within a week of infection and which remain in circulation often for several months after an infection has resolved.

The results showed that the *Babesia* genus-specific FISH test did not detect *Babesia* parasites in the blood of 32 USA patients who had IFA antibody titers indicative of probable active infection. This finding can be caused by sequestration of parasites in the microvasculature resulting in fewer parasites circulating in the peripheral blood or alternatively patients with recently resolved infections who still have high levels of antibodies in their circulation.

Our findings also showed that only 86% of patients positive in the *Babesia* genus-specific FISH test in the USA were positive in IFA tests for *B. microti* or *B. duncani.* Other studies in the USA have also found patients who were positive by direct nucleic acid-based testing and yet negative in IFA tests for babesiosis [20,31]. Very early infections before detectable antibodies are formed, immune evasion mechanisms in *Babesia* that suppress antibody levels, coinfections with tick-borne *Borrelia* that are known to suppress immune responses [28,31] and infection with *Babesia* species other than *B. microti* and *B. duncani* are possible causes for such findings. Relatively high IgM IFA antibody titers and either negative or low IgG titers of in all of the 31 FISH positive and IFA positive patients in our study are consistent with an early stage of infection when parasites are present in peripheral blood at ≥58 parasites per μL in the patients. Two of these patients had IgM antibodies with an IFA titer of 1:160 against *B. microti* and IgG antibodies with IFA titers of 1:320 and 1:160 against *B. duncani*. A resolved or resolving first infection with *B. duncani* followed by a more recent second infection with *B. microti* may explain the IFA findings in these two patients. Some instances of cross-reactions between *B. divergens* and *B. venatorum* as well as the bovine parasites *Babesia argentina* and *Babesia bigemina* in IFA tests have been reported [32,33]. Cross-reactive antibodies between *B. duncani* and *B. microti* or a different *Babesia* species in the two patients is a possibility but would appear unlikely in view of the high antibody titers against both *B. duncani* and *B. microti* in the two patients.

These findings illustrate the advantages of the parallel use of IFA and the *Babesia* genus-specific FISH tests, two complementary tests that detect species-specific anti-*Babesia* antibodies and *Babesia* parasites, respectively, in patients with suspected babesiosis. A set of patients with active infections are identified as positive in both tests. However the two tests also uniquely identify different sets of patients for the likely reasons discussed above.

Notwithstanding the possibility of patients acquiring infections during inter-state travel, our findings suggest that, although *B. duncani* infections are more common in the West coast state of California, *B. duncani* is also a significant cause of babesiosis in the Mid-West and East coast states. The only patient from Puerto Rico examined also had a *B. duncani* positive IFA test. Other recent IFA data also suggest that *B. duncani* is more widespread in the USA [28,31,34] than previously recognized [5,7,22]. Human babesiosis caused by *B. duncani* has also been recently found to be widely distributed in Canada [8]. While the occurrence of human babesiosis had not been extensively studied in Australia, there is evidence suggesting the indigenous transmission of *B. duncani* and *B. microti* in Australia [35]. Our findings now show that babesiosis caused by *B. duncani* and *B. microti* is found throughout continental Australia. *Babesia microti, B. divergens, B. venatorum* are considered to be predominantly responsible for human babesiosis in Europe, though infection with *B. duncani* has been reported [36]. Our findings now show that *B. duncani* is a common cause of babesiosis in Ukraine. We also detected babesiosis caused by *B. duncani* in one patient from India and another patient from Antigua and Barbuda in the Caribbean. Hence, our findings suggest that babesiosis due to *B. duncani* may be more globally prevalent than presently recognized.

The present study demonstrates that a test panel composed of the two IFA tests for *B. duncani* and *B. microti* and the *Babesia* genus-specific FISH test identifies more patients with exposure to babesiosis than the use of the two sets of tests separately, and this is probably the result of different “window” detection periods for the two types of tests. The panel approach for diagnosing babesiosis also provided information on the infecting *Babesia* species. Identifying the *Babesia* species causing infections in patients may be important clinically, because observations in a patient [37] and animal models [15,38] suggest that *B. duncani* may be more pathogenic than *B. microti*.

## Figures and Tables

**Table 1:** USA/Puerto Rico clinical samples.

Test Result	Number of Positive Samples (% of Total)	Conclusion on Babesiosis Status
FISH +ve and IFA −ve	5 (2.0%)	Active infection
FISH +ve and IFA +ve	31 (12.4%)	Active infection
FISH −ve and IFA +ve with IgG titer ≥1:160	32 (12.9%)	Probable active infection
FISH −ve and IFA +ve with IgG titer ≤1:80	21 (8.4%)	Probable resolved infection
Total positive in all tests	89 (35.7%)	Exposure to *Babesia*
Total negative in all tests	160 (64.3%)	No exposure to *Babesia*
FISH +ve and/or IFA +ve with IgG titer ≥1:160	68 (27.3%)	Active infection or probable active infection

+ve = positive; −ve = negative.

**Table 2:** IFA test results with the 36 *Babesia* genus FISH test positive samples from the USA.

IFA Test Species	No. of FISH +ve Samples	Antibody Class and Titer	
		IgM	IgG
*B. microti*	2	1:160	negative
	1*	1:160 Bm	1:320 Bd
	1*	1:160 Bm	1:160 Bd
	1	1:160	1:80
	5	1:80	negative
*B. duncani*	1	1:160	1:40
	1	1:160	1:320
	1	1:80	1:160
	2	1:40	1:160
	4	1:80	1:80
	3	1:80	1:40
	8	1:80	negative
	1	1:40	1:80
IFA negative	5	negative	negative
Total FISH positive	36		

+ve = positive; Bd—*B. duncani*, Bm—*B. microti*.

**Table 3:** Comparison of diagnostic findings for babesiosis in Australia, Europe and the USA.

Region/Country and Number of Samples		FISH +ve and *B. Microti* IFA +ve	FISH −ve and *B. Microti* IFA +ve	FISH +ve and *B. Duncani* IFA +ve	FISH −ve and *B. Duncani* IFA +ve	FISH +ve and IFA −ve	Total FISH or IFA +ve (% of Samples)
Australia	49	2	1	1	10	1	15 (30.6%)
Europe	89	1	2	0	8	7	18 (20.2%)
USA	249	10	14	21	39	5	89 (35.7%)
All	387	13	17	22	57	13	122 (31.5%)

+ve = positive; −ve = negative.

## Supplementary Materials

The following are available online at https://www.mdpi.com/2075-4418/10/10/761/s1: Figure S1. Results of FISH and IFA tests on patient samples from the US states and Puerto Rico; Figure S2. Results of FISH and IFA tests on patient samples from Australia, Europe and other countries.
